# Nutritional quality of children’s menus in restaurants: does cuisine type matter?

**DOI:** 10.1017/S1368980023000344

**Published:** 2023-07

**Authors:** Gina SA Trapp, Natasha Reid, Siobhan Hickling, Alexia Bivoltsis, Joelie Mandzufas, Justine Howard

**Affiliations:** 1 Telethon Kids Institute, Perth Children’s Hospital, 15 Hospital Avenue, Nedlands, WA 6009, Australia; 2 School of Population and Global Health, The University of Western Australia, Nedlands, WA, Australia

**Keywords:** Kids’ menus, Children’s menus, Restaurants, Children, Nutritional quality

## Abstract

**Objective:**

It is unknown whether the nutritional quality of children’s menus varies depending on the cuisine type. This study aimed to investigate differences in the nutritional quality of children’s menus by cuisine type in restaurants located in Perth, Western Australia (WA).

**Design:**

Cross-sectional study

**Setting:**

Perth, WA.

**Participants:**

Children’s menus (*n* 139) from the five most prevalent restaurant cuisine types in Perth (i.e. Chinese, Modern Australian, Italian, Indian and Japanese) were assessed using the Children’s Menu Assessment Tool (CMAT; range -5–21 with lower scores denoting lower nutritional quality) and the Food Traffic Light system, evaluated against Healthy Options WA Food and Nutrition Policy recommendations. Non-parametric ANOVA was used to test for a significant difference in total CMAT scores among cuisine types.

**Results:**

Total CMAT scores were low for all cuisine types (range -2–5), with a significant difference between cuisine types (Kruskal–Wallis *H* = 58·8, *P* < 0·001). The highest total CMAT score by cuisine type was Modern Australian (mean = 2·27, sd = 1·41) followed by Italian (mean = 2·02, sd = 1·02), Japanese (mean = 1·80, sd = 2·39), Indian (mean = 0·30, sd = 0·97) and Chinese (mean = 0·07, sd = 0·83). When using the Food Traffic Light for assessment, Japanese cuisine had the highest percentage of green food items (44 %), followed by Italian (42 %), Modern Australian (38 %), Indian (17 %) and Chinese (14 %).

**Conclusions:**

Overall, the nutritional quality of children’s menus was poor regardless of cuisine type. However, children’s menus from Japanese, Italian and Modern Australian restaurants scored better in terms of nutritional quality than children’s menus from Chinese and Indian restaurants.

Childhood overweight and obesity leads to poor health outcomes in childhood and adult life and is a significant public health concern^(1,2)^. Regularly eating out can increase the risk of childhood overweight and obesity due to the larger portion sizes and poorer nutritional quality of meals served at restaurants compared to home-prepared meals^(3)^. Indeed, children consume more energy and fat when eating food away from the home^(4)^, contributing to overweight or obesity^(5)^. Many restaurants offer children’s menus which contain food and beverage items that are specifically targeted towards children (usually 12 years and under). Children’s menus often lack healthy choices^(6)^, with common foods like deep-fried fish or chicken nuggets and chips and sugar-sweetened beverages often bundled in as the default drink^(7)^. Whilst children’s meals offered in restaurants have been shown to have large portion sizes, high total energy and a high saturated fat content^(8)^, it is unknown whether the nutritional quality of children’s meals served at restaurants differs by cuisine type.

With Australia being one of the most culturally diverse countries in the world^(9)^, the availability of restaurants from varying cuisines is high. Indeed, market share research indicates that the top 10 international food cuisines Australians eat out at most often are Chinese, Italian, Thai, Indian, Mexican, Japanese, Greek, Middle Eastern, Lebanese and French^(10)^. Different cuisine types and the dietary patterns they are made up of have been shown to influence the risk of overweight and obesity. For example, the Mediterranean dietary pattern is characterised by being rich in fruits, vegetables, whole grains, olive oil and low consumption of meat^(11)^. It is associated with reducing cardiovascular risk^(12)^, with a potential function in deterring overweight and obesity^(11)^. The Japanese dietary pattern is characterised by large amounts of fish, soy products, seaweed, vegetables and green tea^(13)^. It has been associated with reduced CVD risk^(13)^. By comparison, a western dietary pattern is characterised by processed foods that are high in added sugar, salt and fat increasing the risk of overweight and obesity, and other chronic health conditions^(14)^.

Differences in the nutritional quality of children’s menus across restaurants of different cuisine type in Australia are unknown. In Japan, a study assessed 438 children’s meals from 42 chain restaurant brands and found food items on Japanese-style children’s menus were lower in fat but higher in salt than the Western-style children’s menus^(15)^. Understanding the nutritional quality of children’s menus from different cuisine types is important given Australians are spending a large proportion of the household income on eating outside the home^(16)^, and there is a high diversity of available cuisine types^(10)^. It would provide valuable insights for parents and caregivers seeking to make healthier choices when eating outside the home and would help inform targeted interventions and policies aimed at improving the healthiness of children’s menus. The aim of this study, therefore, was to investigate whether the nutritional quality of children’s menus differed by cuisine type in restaurants located in Perth, Western Australia (WA).

## Methods

The locations of all restaurants (defined as an establishment that sells food and beverages to customers on the premises, where table service and dinner is available) across Perth, WA, were sourced from each Local Government in 2018–2019. Quick-service style restaurants were excluded. The five most prevalent restaurant cuisine types were Chinese (*n* 251), Modern Australian (*n* 186), Italian (*n* 182), Indian (*n* 170) and Japanese (*n* 108). We aimed to achieve a subsample of 200 children’s menus (i.e. 40 from each of the five selected cuisines). Restaurants within these cuisine types were selected at random and verified using online sources to determine if they were operational and provided a children’s menu. If there was no children’s menu available online, restaurants were contacted by email, social media or telephone in June – July 2021 to obtain their evening children’s menu until a maximum of 40 from each cuisine type was achieved.

The nutritional quality of children’s menus was assessed using the Children’s Menu Assessment Tool (CMAT)^(6)^ and the Food Traffic Light system^(17)^ based on the Healthy Options WA Food and Nutrition Policy^(18)^. The CMAT has 29 items, from which only 21 items are included in the total score which ranges from -5–21^(6)^. A higher score indicates more healthier food and beverage options are available^(6)^. The Food Traffic Light system classifies food and beverage items as green, amber or red. Items classified as ‘green’ are from the five core food groups, recommended for everyday consumption and a good source of nutrients^(17)^. Items classified as ‘amber’ have a degree of nutritional value but may contain moderate amounts of fats, excess sugar and salt^(17)^. Items classified as ‘red’ are often high in energy, fat, sugar salt and represent discretionary items in the Australian Guide to Healthy Eating^(17)^. Each food and drink item on the children’s menus was assessed separately (i.e. each main, side, any additional side dishes that cost extra, dessert, drinks). If a meal contained fish with chips, then chips was considered a side and assessed separately. The proportion of food and beverages on each children’s menu from the red, amber and green categories was calculated and compared to the Healthy Options WA Food and Nutrition Policy recommendations (i.e. a minimum of 50 % of items offered are green food and drinks, no more than 20 % of foods offered are red with no red drinks, the remaining items offered may be amber food and drinks)^(18)^. Currently, this policy is only mandatory in WA Health Department run entities, but is encouraged in other settings^(18)^.

Following a Kolmogorov–Smirnov test (data were not normally distributed), a non-parametric Kruskal–Wallis ANOVA test assessed whether there was a significant difference among cuisine types in the total CMAT scores from children’s menus. The proportions of green, amber and red items on each children’s menu were assessed and compared against the recommended Healthy Options WA Food and Nutrition Policy (i.e. a minimum of 50 % of items offered are green food and drinks, no more than 20 % of foods offered are red with no red drinks, the remainder may be amber food and drinks).

## Results

Sixty Modern Australian restaurants, 61 Italian restaurants and 110 Indian restaurants were assessed to achieve our target of 40 children’s menus. Only 200 of the 251 Chinese restaurants were operational, and only 14 had a children’s menu. Only 76 of the 108 Japanese restaurants were operational, and only 5 had a children’s menu. A total of 139 children’s menus were collected.

Modern Australian restaurants had the highest proportion of children’s menus available (67 %), followed by Italian (66 %), Indian (36 %), Chinese and Japanese (7 %). The three dishes most frequently present on children’s menus by cuisine type included Chinese (fish and chips, chicken nuggets and chips, chips); Modern Australian (fish and chips, pasta, chicken nuggets and chips); Italian (pasta, pizza, chicken nuggets and chips); Indian (curry and rice/chips, chicken nuggets and chips, fish and chips); and Japanese (chicken and chips, fish and chips, sushi).

Table 1 shows summary statistics for total CMAT scores across the five cuisine types. Total CMAT scores were very low across all cuisine types (range -2–5), but a significant difference overall was found (Kruskal–Wallis *H* = 58·8, *P* < 0·001). The highest CMAT score by cuisine type was Modern Australian (mean = 2·27, sd = 1·41) followed by Italian (mean = 2·02, sd = 1·02), Japanese (mean = 1·80, sd = 2·39), Indian (mean = 0·30, sd = 0·97) and Chinese (mean = 0·07, sd = 0·83).

**Table 1:** Summary statistics for the total scores obtained from the Children’s Menu Assessment Tool for children’s menus from each restaurant cuisine type in Perth, Western Australia

	Chinese (*n* 14)	Modern Australian (*n* 40)	Italian (*n* 40)	Indian (*n* 40)	Japanese (*n* 5)
Range	−2·00–2·00	−1·00–5·00	0·00–5·00	−1·00–4·00	−1·00–5·00
Mean	0·07	2·27	2·02	0·30	1·80
sd	0·83	1·41	1·02	0·97	2·39

Table 2 shows results from the Food Traffic Light system analyses. Only one Japanese (20 %) and one Italian menu (3 %) met the recommended Healthy Options WA Food and Nutrition Policy of providing a minimum of 50 % green food and drinks, no more than 20 % red foods, no red drinks and the remainder being amber food and drinks. No Indian, Chinese or Modern Australian children’s menus met the recommended Healthy Options WA Food and Nutrition Policy. Overall, Japanese children’s menus had the highest percentage of green food items (44 %), followed by Italian (42 %), Modern Australian (38 %), Indian (17 %) and Chinese (14 %).

**Table 2:** The number and percentage of green, amber or red food and drink items on children’s menus assessed via the Food Traffic Light system for each restaurant cuisine type in Perth, Western Australia

	Green food items *n*	%	Amber food items *n*	%	Red food items *n*	%	Green drinks *n*	%	Amber drinks *n*	%	Red drinks *n*	%	Children’s menus that met policy requirements *n*	%*
Chinese (*n* 14)	8	14	0		49	86	0		0		4	100	0	0
Modern Australian (*n* 40)	103	38	10	4	158	58	1	3	10	31	21	66	0	0
Italian (*n* 40)	136	42	6	2	180	56	6	26	5	22	12	52	1	3
Indian (*n* 40)	44	17	11	4	200	78	1	17	1	17	4	67	0	0
Japanese (*n* 5)	14	44	0		18	56	0		1	33	2	67	1	20

* Healthy options Western Australia food and nutrition policy (i.e. a minimum of 50 % green food and drinks, no more than 20 % red foods, no red drinks and the remainder being amber food and drinks).

## Discussion

This study found that items offered on children’s menus were nutritionally poor, regardless of cuisine type. Most children’s meals in our study featured high-fat foods (i.e. chips) and sugar-sweetened beverages, with core food groups largely ignored. The lack of core food groups indicates that children’s menus may be low in key nutrients such as calcium, vitamin C, iron and fibre^(19)^. Poor nutritional quality of children’s menus has been consistently demonstrated in several countries (e.g. the US, UK, Ireland, Germany and Canada) and within cafes, restaurants and fast-food outlets^(20)^. Currently, it is unclear why children’s meals are lacking in nutritional quality. Children may be afraid or uncertain to try new or different foods and prefer to eat familiar foods or see eating out as a treat, and parents may therefore take the path of least resistance, driving demand. Ultimately, food business owners will shape their menu to maximise profit. However, with associated changes to pricing and menu layout, children and parents are receptive to healthier items on children’s menus^(21)^, and restaurant revenue is not compromised^(22)^.

Whilst the nutritional quality of children’s menus was poor for all five cuisine types examined, some were better than others. For example, children’s menus from Italian, Japanese and Modern Australian restaurants performed best overall. Most Italian restaurants offered pizza or pasta with tomato-based sauces, often combined with a side of salad or vegetables. Our study found that Chinese and Indian cuisine types scored lowest nutritionally, with fewer healthy items available on the children’s menus. Food items offered were predominantly deep-fried fish or chicken nuggets with chips, or chips alone, which is not consistent with the typical food items of the cuisine type.

Given that healthful food for children aged two and older is the same as for adults, with age-appropriate adjustments in texture and portion size, a recent position statement from the Society for Nutrition Education and Behaviour posits that children can, and should, eat the same foods as adults^(23)^. If restaurant children’s menus comprised smaller portion sizes of main menu items at a reduced price for children, they would be more likely to maintain cultural integrity and increase the amount, variety and potentially the nutritional quality of foods offered to children. Future research should explore whether the nutritional quality and food purchasing decisions differ when restaurants offer separate children’s menus with ‘traditional’ unhealthy options, compared with the option to order from the main menu with a reduced price and portion size.

The findings from this study highlight that interventions and policies aimed at improving the healthiness of restaurant children’s menus are needed across all cuisine types. Several initiatives in the United States and one in Australia encourage restaurants to offer healthy options on children’s menus and to improve their overall healthfulness,^(24)^ for example, the US National Kids LiveWell Program, The Best Food for Families, Infants and Toddlers intervention and the ‘Healthy Kids Menu’ initiative undertaken by South Australia Health and Health and Wellbeing Queensland^(20)^.

This appears to be the first study to assess the nutritional quality of children’s menus from restaurants of different cuisine type. Future research should explore the nutritional quality of children’s menus from a wider range of cuisine types and incorporate other methods of nutritional assessment (e.g. laboratory assessment of total energy, macro and micronutrients). It is important to note that owing to the limited descriptive information available on the children’s menus, some assumptions were made in our study about cooking methods and ingredients. Therefore, it is possible that some food items may have been misclassified. Furthermore, it was not possible to consider food weight based on menu information only, which could alter the overall nutritional quality of meals depending on the actual weights of green, amber and red foods.

Overall, our study found the nutritional quality of children’s menus offered at restaurants in Perth, WA, was poor regardless of cuisine type; however, some cuisines performed better than others. Interventions to improve the nutritional quality of food and beverages offered to children at restaurants are needed.
